# The Dementia Rating Scale (DRS) in the diagnosis of vascular dementia

**DOI:** 10.1590/S1980-57642008DN10300010

**Published:** 2007

**Authors:** Cláudia Sellitto Porto, Paulo Caramelli, Ricardo Nitrini

**Affiliations:** 1 PhD, Behavioral and Cognitive Neurology Unit, Department of Neurology of the University of São Paulo School of Medicine and Cognitive Disorders Reference Center (CEREDIC), Hospital das Clínicas of the University of São Paulo School of Medicine, São Paulo, SP, Brazil.; 2 MD, PhD, Behavioral and Cognitive Neurology Unit, Department of Internal Medicine, Faculty of Medicine, Federal University of Minas Gerais, Belo Horizonte, MG, Brazil.; 3 MD, PhD, Behavioral and Cognitive Neurology Unit, Department of Neurology of the University of São Paulo School of Medicine, and Cognitive Disorders Reference Center (CEREDIC), Hospital das Clínicas of the University of São Paulo School of Medicine, São Paulo, SP, Brazil.

**Keywords:** dementia, neuropsychological tests, cognitive disorders, Alzheimer disease, vascular dementia

## Abstract

The Dementia Rating Scale (DRS) is considered a very useful instrument to assess patients with dementia. The tasks are grouped into five subscales, each one evaluating different cognitive areas, namely: Attention, Initiation/Perseveration (I/P), Construction, Conceptualization and Memory.

**Objective:**

To verify the ability of the DRS in discriminating vascular dementia (VaD) patients from healthy controls and VaD from Alzheimer disease (AD) patients.

**Methods:**

The DRS was applied to 68 patients with mild dementia (12 with VaD and 56 with AD) and 60 healthy controls. The clinical diagnosis was made by two neurologists based on the patients´ history, laboratory and neuroimaging results and neuropsychological tests.

**Results:**

In the comparison between VaD patients and controls, the subscales I/P, Memory, Conceptualization and Attention were those displaying best discrimination between the two groups. The cutoff <124 yielded 93.3% of sensitivity and 91.7% of specificity for the diagnosis of VaD. Only the I/P subscale differentiated VaD from AD patients.

**Conclusions:**

The DRS was found to be a useful instrument to discriminate VaD patients from controls. VaD patients showed worse performance in tasks of executive functions than AD patients. Executive dysfunction, evaluated through the I/P subscale of the DRS, might be useful in differentiating between VaD and AD patients.

## Abstract (pt)

A Escala de Avaliação de Demência (Dementia Rating Scale – DRS) é considerada um instrumento de grande valor para avaliação de pacientes com demência. As tarefas estão agrupadas em cinco subescalas, as quais avaliam diferentes domínios cognitivos: Atenção, Iniciativa/Perseveração (I/P), Construção, Conceituação e Memória.

**Objetivo:**

Verificar a capacidade da DRS em discriminar pacientes com demência vascular (DV) de controles saudáveis e pacientes com DV de pacientes com doença de Alzheimer (DA).

**Métodos:**

A DRS foi administrada a 68 pacientes com demência leve (12 com DV e 56 com DA) e a 60 indivíduos controles saudáveis do ponto de vista cognitivo. Os diagnósticos de DV e de DA foram realizados por dois neurologistas baseando-se na história clínica, em exames laboratoriais e de neuroimagem e em testes neuropsicológicos.

**Resultados:**

Na comparação de pacientes com DV e controles, as subescalas I/P, Memória, Conceituação e Atenção foram as que melhor diferenciaram os dois grupos. A nota de corte <124 demonstrou sensibilidade de 93,3% e 91,7% de especificidade para o diagnóstico de DV. Em relação à comparação de pacientes com DV e DA, apenas a subescala I/P apresentou significância estatística na diferenciação dos dois grupos.

**Conclusões:**

A DRS mostrou ser um instrumento útil na discriminação entre pacientes com DV e controles. Pacientes com DV demonstraram pior desempenho em tarefas de funções executivas que pacientes com DA. Disfunção executiva, avaliada através das tarefas da subescala I/P, pode ser útil na diferenciação entre pacientes com DV e DA.

The Dementia Rating Scale (DRS)^1,2^ is a measure of general cognitive status and has been used both in clinical practice and research. The scale includes 36 tasks which are grouped into five subscales assessing different cognitive domains, namely: Attention, Initiation/Perseveration (I/P), Construction, Conceptualization and Memory.

The value of the DRS has been reaffirmed by several studies that have cited the use of this scale in the diagnosis and discrimination of patients with Alzheimer disease (AD) from those with other forms of dementia. Previous investigators have reported that the DRS is able to differentiate patients with AD from cognitively healthy controls,^3,4^ as well as AD from dementia associated with Parkinson´s disease,^5^ Huntington's disease from AD,^6^ and patients with vascular dementia (VaD) from patients with AD.^7,8^

The main objective of this work is to verify the ability of the DRS to discriminate VaD patients from controls, and VaD from AD patients.

## Methods

The study involved 68 patients (39 women and 29 men), aged 54 to 84 years (mean=72.35±7.78), with schooling ranging from 3 to 17 years (mean=9.40±4.78), attended by members of the Behavioral and Cognitive Neurology Unit of the Department of Neurology at the University of São Paulo School of Medicine, in Brazil. All patients were submitted to appropriate laboratory tests and to structural neuroimaging (computed tomography (CT) or magnetic resonance (MR) of the skull). Moreover, they were submitted to a comprehensive neuropsychological evaluation, which included the following tests: the Mini-Mental State Examination (MMSE),^9,10^ the Brief Cognitive Screening Battery (BCSB),^11^ visual and verbal memory tests (subtest Visual Reproduction of the Wechsler Memory Scale – Revised (WMS-R),^12^ Rey Complex Figure – delayed recall,^13^ subtest Logical Memory (WMS-R),^12^ Rey Auditory Verbal Learning Test (RAVLT),^14^ constructive abilities (subtest Block Design –Wechsler Adult Intelligence Scale (WAIS),^15^ Rey Complex Figure copy,^13^ visual perception (Hooper Visual Organization Test^16^ and Raven´s Progressive Matrices,^17^ language (Boston Naming Test),^18^ and executive functions (Trail Making Test versions A and B,^19^ Stroop Test,^19^ Wisconsin Card Sorting Test (WCST)^19^ and phonemic verbal fluency (F.A.S.).^19^ Information on performance in daily life activities was obtained through the Pfeffer Functional Activities Questionnaire,^20^ which was applied to an informant.

The clinical diagnosis of mild dementia was based on the criteria of the Diagnostic and Statistical Manual of Mental Disorders, Third Edition, revised (DSM-III-R)^21^ and was made by two neurologists (PC and RN), who were blind to DRS and BCSB results, and based on the patients’ history, laboratory and neuroimaging results, MMSE scores and on results of the following neuropsychological tasks: constructive abilities (Block Design (WAIS), memory (Rey Auditory Verbal Learning Test (RAVLT) – sum of scores from trials 1 to 5 and the number of words recalled after 30 minutes), language (Boston Naming Test), executive functions (phonemic verbal fluency and Trail Making Test (versions A and B)).

The AD group was composed of 56 individuals, aged 54 to 84 years (mean=72.98±7.43), with schooling ranging from 3 to 17 years (mean=9.62±4.68), comprising 35 women and 21 men. The diagnosis of probable AD was based on the criteria of the National Institute of Neurological Disorders and Communicative Disorders and Stroke-Alzheimer Disease and Related Disorders Association (NINCDS-ADRDA).^22^

Twelve patients were included in the VaD group, aged 54 to 80 years (mean=69.41±8.99), with schooling ranging from 4 to 16 years (mean=8.33±5.30), comprising 4 women and 8 men (nine cases of subcortical VaD and three cases of multiple infarct dementia). Eleven patients were submitted to MRI of the skull and one to CT. The diagnosis of probable VaD was based on the criteria of the National Institute of Neurological Disorders and Stroke – Association Internationale pour la Recherche et l´Enseignement en Neurosciences (NINDS- AIREN).^23^

The control group (60 subjects; mean age=68.90±7.48; mean schooling=10.72±4.74; 42 women and 18 men) was composed of spouses or consorts of the patients, or volunteers from the community, with no memory disorders and who were self-sufficient in terms of daily life activities. Subjects with neurological disease, history of alcoholism, depression, or any other psychiatric disorder, non-corrected visual or auditory disorders, motor disorders, or users of psychotropic drugs that could affect cognitive functions were excluded. Chronic diseases such as arterial hypertension, diabetes mellitus and cardiac disorders, if adequately controlled, were not criteria for exclusion. All controls were submitted to the MMSE, the BCSB and to the Memory Complaint Questionnaire (MAC-Q) (24) or to the Informant Questionnaire on Cognitive Decline in the Elderly (IQCODE),^25,26^ administered to an informant.

The Portuguese version of the DRS^17^ was administered to all patients and controls. The tasks are presented in a fixed order, as recommended by the author, and only the Attention tests are not grouped in a sequence, as they also serve as distractors for the Memory subscale. Within each subscale, the most difficult tests were presented in first and second, and if performed well, subsequent items of the subscale were automatically scored as having been performed correctly. The advantage of this procedure is that it shortens total testing time for individuals who are relatively intact.

The number of points scored for the correct response varies in accordance with the tasks, while the total number of points in each subscale provides a partial score for that subscale. The partial scores are: Attention, 37 points; Initiation/Perseveration, 37 points; Construction, 6 points; Conceptualization, 39 points; and Memory, 25 points. The maximum possible score on the DRS is 144 points.

In the two groups studied, the scale was applied individually in a single session. The time of application for the group of patients was, on average, 40 minutes, and for the control group, from 20 to 30 minutes.

The study was approved by the Research end Ethics Committee of Hospital das Clínicas of the University of São Paulo School of Medicine. All subjects who agreed to participate signed a written informed consent.

### Statistical analysis

In order to evaluate associations between the categorical variables and the results, the Pearson Chi-Squared test was performed. When the variables were continuous, the comparisons were made for two samples by the Mann-Whitney test, and for more than two, by the Kruskall-Wallis test.

Sensitivity and specificity calculations were performed for each subscale and for the total scale. The cutoff score, calculated through ROC (*receiver operator characteristics*) curves, was defined as the value presenting the best relationship between sensitivity and specificity.

Alpha risk was considered to be less than or equal to 5% for type 1 error and beta risk greater than or equal to 20% for type II error.

All statistical analysis was carried out using the program Statistical Package for the Social Sciences (SPSS), version 10.0.

## Results

There were no statistically significant differences between controls and both patient groups in relation to schooling (p=0.213) and gender (p=0.055), but there was a significant difference in relation to age (p=0.011). A statistically significant difference was found between mean total DRS scores of controls and both patients' groups (Table 1).

**Table 1:** Performance of patients with VaD and controls, and VaD and AD, DRS total and subscales.

	VaD	controls	p	AD	p
N	12	60		56	
Total					
Mean (SD)	110.1 (11.0)	136.2 (6.3)	<0.0001	113.8 (12.4)	0.314
Attention					
Mean (SD)	35.2 (0.6)	35.7 (1.3)	0.021	34.9 (1.6)	0.818
I/P Mean (SD)	24.6 (4.2)	35.1 (1.9)	<0.001	29.1 (5.8)	0.010
Construction					
Mean (SD)	5.8 (0.5)	5.8 (0.3)	0.150	5.6 (1.0)	0.549
Conceptualization Mean (SD)	28.2 (4.8)	34.5 (4.0)	<0.001	29.4 (6.0)	0.600
Memory Mean (SD)	16.3 (4.0)	24.0 (1.5)	<0.001	14.7 (3.9)	0.325

N, subjects; I/P, initiation/perseveration; SD, standard deviation; p<0.05.

### VaD patients and controls

**Figure 1:**
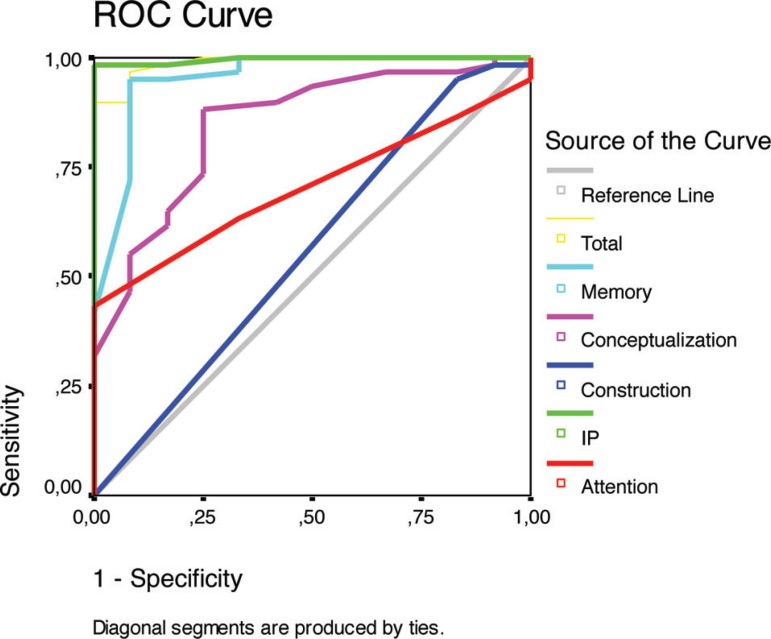
ROC curves of the DRS total score and overall subscales between VaD patients and controls.

In the analysis of the mean total scores on the total scale and each subscale, the I/P subscale (p<0.001) as well as Memory (p<0.001), Conceptualization (p<0.001) and Attention (p=0.021) subscales differentiated VaD from controls. The scores in the Construction subscale (p=0.150) were not significantly different between the two groups. The same phenomenon occurred in the analysis of the areas under curves obtained through the ROC curves (Figure 1) (Table 2).

**Table 2:** Areas under the curves, cutoff, sensitivity and specificity for the DRS between VaD patients and controls.

DRS	AUC (SE)	Maximum points	cutoff *	Sensitivity	Specificity
Total	0.989±0.010	144	<124	93.3	91.7
Attention	0.704±0.063	37	<34	63.3	66.7
I/P	0.996±0.005	37	<29	98.3	83.3
Construction	0.558±0.097	6	<6	95.0	16.7
Conceptualization	0.848±0.059	39	<31	81.7	75.0
Memory	0.953±0.036	25	<21	95.0	91.7

DRS, Dementia Rating Scale; AUC, area under curve; SE, standard error; I/P, initiation/perseveration;

* individuals with score below the cutoff score are impaired.

### VaD and AD patients

The performance of the AD group on the DRS was compared to the VaD patients’ scores (Table 1).

In the comparison between VaD and AD patients, only the I/P subscale was able to significantly differentiate between the two groups (p=0.010) (AUC=0.739±0.064; p=0.010).

## Discussion

In the present study, the DRS was able to accurately discriminate VaD patients from controls, while only the I/P subscale differentiated VaD from AD patients.

In the discrimination between VaD patients and control individuals, the cutoff score <124 in the DRS showed good sensitivity (93.3%) and specificity (91.7%) values.

Both in the analysis of the areas under the curves (AUC) and comparison between the means scores of the two groups, I/P, Memory, Conceptualization and Attention subscales also allowed good discrimination between VaD patients and controls.

The Memory subscale differentiated VaD patients from normal elderly. Lukatela et al.^8^ verified, in their study comparing DRS scores in VaD, AD and controls, that the group with AD and the group with VaD presented significant impairment in comparison to the control group. Price et al.^29^ concluded that tests of executive control and memory, along with neuroimaging evidence of involvement of around one-fourth of the cerebral white matter as measured by the Leukoaraiosis Scale, may be sufficient for the diagnosis of subcortical VaD.

The results of the Inasaridze et al.^34^ study demonstrated that attentional deficits are characteristic of VaD. Impaired attention was also observed in other studies,^35,36^ a feature in agreement with our results on the Attention subscale of the DRS.

The Construction subscale was not able to discriminate VaD patients from controls. This finding seems to be in contrast with the work of Lukatela et al.^8^ in which VaD patients showed greater impairment on this subscale compared to AD individuals. According to these authors, the results demonstrated that problems in simple graphomotor construction and coordination are more pronounced in VaD than in AD. Perhaps due to the small number of patients in our series, our results differ from those published in the literature.

Patients with VaD and controls also showed different performances on the Conceptualization subscale in the present study. Giovannetti et al.^33^ investigated different mechanisms that may underlie deficits in verbal concept formation among patients with AD and ischemic VaD. The test utilized by the authors was the Wechsler Adult Intelligence Scale – Revised (WAIS-R). The Similarities subtest, which contains similar tasks as the Conceptualization subscale, did not differentiate between the two groups. Nonetheless, AD patients produced a greater proportion of very vague superordinate concepts for the word pairs (for example: dog/lion: “they´re alive”) while the errors produced by VaD patients demonstrated an inability to provide a superordinate concept for the same word pairs (for example: dog/lion: “the lion roars and the dog barks”). The errors produced by VaD patients showed impairment in concept formation associated with deficits in executive systems necessary to monitor responses and to sustain mental set. The AD patients´ errors were associated with measures of delayed recognition memory and semantic intrusion errors, indicating that the deficit of concept formation appears to be secondary to impaired verbal response selection.

The comparison between the performances of VaD and AD patients on the DRS showed that only the I/P subscale was able to differentiate between the two groups.

Similar results were reported by two independent investigations. Kertesz and Clydesdale^7^ compared AD and VaD patients performances on the DRS. VaD patients were significantly worse on motor performance subtests of the I/P subscale than AD patients. The authors concluded that these subtests might be useful in discriminating between VaD and AD. In the above-mentioned Lukatela et al. study,^8^ VaD patients with multiple infarcts demonstrated significantly lower scores on the I/P subscale than AD patients.

The I/P subscale of DRS is composed by verbal fluency for semantic categories (supermarket items), double simultaneous hand movements and design copy tasks. These two latter tasks evaluate bimanual coordination and motor perseveration, which are recognized to be associated to frontal lobes deficits. According to some authors,^27-31^ the executive dysfunction might serve as diagnostic marker for VaD, especially for the subcortical subtype. Villardita^32^ verified that attention processes, planning and fine motor coordination tasks were more severely impaired in VaD than in AD patients, concluding that these disturbances resemble some of those occurring in frontal lobe syndromes. VaD patients were significantly disadvantaged in executive functions which include planning and sequencing, speed of mental processing, performance on unstructured tasks, and also attention.

In conclusion, the DRS in the present study proved a useful instrument to discriminate between VaD patients and controls. Our results suggest that executive dysfunction, evaluated through the I/P subscale tasks, is helpful in differentiating VaD from AD patients. Further studies involving larger samples of patients are necessary in order to confirm these initial findings.
